# Bone Marrow Mesenchymal Stem Cells' Secretome Exerts Neuroprotective Effects in a Parkinson's Disease Rat Model

**DOI:** 10.3389/fbioe.2019.00294

**Published:** 2019-11-01

**Authors:** Bárbara Mendes-Pinheiro, Sandra I. Anjo, Bruno Manadas, Jorge D. Da Silva, Ana Marote, Leo A. Behie, Fábio G. Teixeira, António J. Salgado

**Affiliations:** ^1^Life and Health Sciences Research Institute (ICVS), School of Medicine, University of Minho, Campus de Gualtar, Braga, Portugal; ^2^ICVS/3B's-PT Government Associate Laboratory, Braga/Guimarães, Portugal; ^3^CNC-Center for Neuroscience and Cell Biology, University of Coimbra, Coimbra, Portugal; ^4^Canada-Research Chair in Biomedical Engineering (Emeritus), Schulich School of Engineering, University of Calgary, Calgary, AB, Canada

**Keywords:** Parkinson's disease, mesenchymal stem cells, secretome, dopamine neurons, neuroprotection

## Abstract

Parkinson's disease (PD) is characterized by a selective loss of dopamine (DA) neurons in the human midbrain causing motor dysfunctions. The exact mechanism behind dopaminergic cell death is still not completely understood and, so far, no cure or neuroprotective treatment for PD is available. Recent studies have brought attention to the variety of bioactive molecules produced by mesenchymal stem cells (MSCs), generally referred to as the secretome. Herein, we evaluated whether human MSCs-bone marrow derived (hBMSCs) secretome would be beneficial in a PD pre-clinical model, when compared directly with cell transplantation of hBMSCs alone. We used a 6-hydroxydpomanie (6-OHDA) rat PD model, and motor behavior was evaluated at different time points after treatments (1, 4, and 7 weeks). The impact of the treatments in the recovery of DA neurons was estimated by determining TH-positive neuronal densities in the *substantia nigra* and fibers in the striatum, respectively, at the end of the behavioral characterization. Furthermore, we determined the effect of the hBMSCs secretome on the neuronal survival of human neural progenitors *in vitro*, and characterized the secretome through proteomic-based approaches. This work demonstrates that the injection of hBMSCs secretome led to the rescue of DA neurons, when compared to transplantation of hBMSCs themselves, which can explain the recovery of secretome-injected animals' behavioral performance in the staircase test. Moreover, we observed that hBMSCs secretome induces higher levels of *in vitro* neuronal differentiation. Finally, the proteomic analysis revealed that hBMSCs secrete important exosome-related molecules, such as those related with the ubiquitin-proteasome and histone systems. Overall, this work provided important insights on the potential use of hBMSCs secretome as a therapeutic tool for PD, and further confirms the importance of the secreted molecules rather than the transplantation of hBMSCs for the observed positive effects. These could be likely through normalization of defective processes in PD, namely proteostasis or altered gene transcription, which lately can lead to neuroprotective effects.

## Introduction

Parkinson's disease (PD) represents the second most common neurodegenerative disorder after Alzheimer's disease, affecting ~1% of the population worldwide over 65 years old (Vos et al., 2017). Its pathogenesis is characterized by the death of dopamine (DA) neurons in the *substantia nigra* pars compacta (SNpc), leading to a decrease of DA levels in the striatum, which consequently causes typical motor dysfunctions, such as tremor ate rest, rigidity, bradykinesia, among others (Przedborski, 2017; Axelsen and Woldbye, 2018). Another important hallmark feature of PD is the presence of Lewy bodies that are abnormal aggregates of proteins enriched in α-synuclein (Axelsen and Woldbye, 2018). Current therapies, such as the administration of DA analogs or deep brain stimulation, are only focused on reducing the symptoms but fail to stop disease progression or to rescue the cells and the neuronal circuit (Anisimov, 2009; Sethi, 2010). On the other hand, stem cell-based therapies have been providing great opportunities to develop innovative strategies for PD therapy (Mahla, 2016). Within a variety of promising cell sources, mesenchymal stem cells (MSCs) have stood out as a valid therapeutic option (Mendes-Pinheiro, 2016). The initial research claimed that the engraftment and differentiation capacity of MSCs was the main responsible mechanism of their therapeutical effects. However, recent studies brought attention to the bioactive molecules produced by MSCs, generally referred to as the secretome (Teixeira et al., 2013; Vizoso et al., 2017). Among these set of factors/molecules released by MSCs we can list the soluble proteins (e.g., cytokines, chemokines, and growth factors), lipids and the extracellular vesicles, that are known for the capacity of promoting cell survival and differentiation, prevent neuronal cell death, protect other cells from oxidative stress or even regulate inflammatory processes (Baraniak and McDevitt, 2010; Teixeira et al., 2013; Marques et al., 2018). Previously, we have already shown that human MSCs-bone marrow derived (hBMSCs) secretome potentiated the increase of tyrosine hydroxylase (TH)-positive in the SNpc and striatum, respectively, which supports the improvements observed in the Parkinsonian animals (Teixeira et al., 2017). In fact, the use of secretome *per se* presents numerous advantages when compared with more conventional stem-cell based applications, regarding manufacturing, storage, handling, their potential as a ready-to-use biologic product and lack of immunosuppression-based adjuvant therapies (Vizoso et al., 2017). For instance, the time and cost of expansion and maintenance of cultured MSCs could be significantly reduced, and the storage can be done for long periods without loss of product potency and quality (Bermudez et al., 2015, 2016; Vizoso et al., 2017). The production in large quantities is possible under controlled laboratory conditions and the biological product could be modified to desired cell-specific effects (McKee and Chaudhry, 2017; Vizoso et al., 2017). Importantly, the use of the secretome derivatives could bypass potential issues associated with cell transplantation including the number of available cells for transplantation and its survival after this procedure, immune compatibility, tumorigenicity, and infection transmission (Tran and Damaser, 2015).

In view of the above, the main objective of this work was to study the efficacy of hBMSCs secretome when compared to the traditional approach in the field, that is hBMSCs transplantation, particularly on DA neurons survival and motor function of a 6-hydroxydopamine (6-OHDA) rat PD model. Here, we demonstrate that hBMSCs secretome was able to minimize the loss of DA neurons and ameliorates the motor deficits of 6-OHDA-lesioned animals. Moreover, we also observed that hBMSCs were able to induce neuronal differentiation *in vitro* and highlighted possible proteins and mechanisms that could mediate the above referred actions.

## Materials and Methods

### Human Bone Marrow Mesenchymal Stem Cells Preparation

hBMSCs (Lonza, Switzerland) were defreeze and plated into T-75 gelatin (0.1%, Sigma, USA)-coated culture flasks with serum-free growth medium (PPRF-msc6) that was prepared as described elsewhere in detail (Jung et al., 2010). The medium changes were done every 3 days, and after reached 80% of confluence, cells were harvested using 0.05% trypsin/EDTA (Invitrogen, USA) and plated again in gelatin-coated flasks at a density of 5,000 cells/cm^2^. For all *in vitro* experiments, hBMSCs from three different donors were used. The hBMSCs used in this work were previously characterized by our lab (Teixeira et al., 2016), being positive for the standard hMSCs markers CD13, CD73, CD90, and CD105, and negative for CD34, CD45, and HLA-DR.

### Secretome Collection and Concentration

The secretome of hBMSCs was collected under the form of conditioned medium in passage 5 (P5) according to protocols already established in our laboratory (Fraga et al., 2013; Teixeira et al., 2016). Briefly, cells were seeded at a density of 5,000 cells/cm^2^ for the *in vivo* experiments and 12,000 cells/cm^2^ for proteomic analysis. After 3 days in culture, the cells were washed three times with PBS without Ca^2+^/Mg^2+^ (Invitrogen), and once with Neurobasal-A medium (TermoFisher Scientific, USA) supplemented with 1% kanamycin (Life Technologies, USA), being incubated with this medium during 24 h. In the next day, the medium comprising the elements secreted by hBMSCs was collected and centrifuged at 1,200 rpm (Megafuge 1.0R, Heraeus, Germany) for 10 min to remove any cell debris. Then, hBMSCs secretome was concentrated (100 ×) by centrifugation using a 5 kDa cut-off concentrator (Vivaspin, GE Healthcare, UK) and frozen at −80°C until used for proteomic analysis and surgical procedures, as previously described (Mendes-Pinheiro et al., 2018).

### Neural Progenitor Cells Growth and Incubation With hBMSCs Secretome

For neuronal differentiation studies, pre-isolated and cryopreserved human neural progenitor cells (hNPCs) were thawed at 37°C and grew as neurospheres in serum-free medium PPRF-h2 (Baghbaderani et al., 2010; Mendes-Pinheiro, 2016). Cells were isolated in respect with the protocols and strict ethical guidelines previously established and approved by the Conjoint Health Research Ethics Board (CHREB, University of Calgary, Canada; ID: E-18786) (Mendez et al., 2002, 2005; Baghbaderani et al., 2010). After 3 days, cells were mechanically triturated to form single cells and cultivated again in fresh medium. After 14–20 days of growth, hNPCs were enzymatically dissociated and plated on 24-well plates coated with poly-D-lysine (100 μg/mL; Sigma) and laminin (10 μg/mL; Sigma) using 50,000 cells per well. The cells were exposed for 5 days to hBMSCs secretome, and supplemented Neurobasal-A medium [differentiation media; 2% B27 (Gibco, USA), 0.05% basic fibroblast growth factor (bFGF; R&D Systems, USA), 1% kanamycin, and 0.5% GlutaMAX (Gibco)] was used as positive control, and Neurobasal-A medium only with 1% kanamycin (basal media) was used as negative control group (Mendes-Pinheiro, 2016).

### Immunocytochemical Staining

hNPCs were fixed in 4% paraformaldehyde (PFA; Merck, Portugal) for 30 min at room temperature (RT), to retain the antigenicity of the target molecules and preserve cell morphology. The permeabilization was done in phosphate buffered saline (PBS) with 0.1% Triton X-100 (PBS-T; Sigma) for 5 min, followed by blockage of non-specific binding sites using PBS with 10% newborn calf serum (NBCS; Biochrom, Germany) for 1 h. hNPCs were then incubated with the following primary antibodies: anti-doublecortin (DCX, rabbit polyclonal IgG, 1:300; ab18723, Abcam, UK) for immature neurons and anti-microtubule associated protein-2 for mature neurons (MAP-2, mouse monoclonal IgG, 1:500; M4403, Sigma), diluted in PBS with 10% NBCS for 1 h at RT. After washing, cells were incubated with the secondary antibodies Alexa Fluor 488 goat anti-rabbit (1:1000; Life Technologies) or Alexa Fluor 594 goat anti-mouse (1:1000; Life Technologies) for 1 h at RT. Nuclei were stained with 4-6-diamidino-2-phenylindole-dhydrochloride (DAPI, 1:1000; Life Technologies) for 10 min at RT. Afterwards, coverslips were mounted on glass slides using immu-mount (Thermo Scientific, UK). Finally, samples were observed using fluorescence microscopy (BX61, Olympus, Germany), being 3 coverslips per condition and 10 representative fields chosen for quantification analysis. The overall proportions of DCX or MAP-2-positive cells in all experiments were pooled and used for comparison between groups, and the quantification was done under blind conditions.

### Subjects and Surgical Procedures

All animal experimentation was performed with the consent to the Portuguese national authority for animal research, Direção Geral de Alimentação e Veterinária (ID: DGAV28421) and Ethical Subcommittee in Life and Health Sciences (SECVS; ID: SECVS-008/2013, University of Minho). We used 8-weeks old *Wistar-Han* male rats (260–300 g; Charles River, Spain) that were caged in pairs with food and water *ad libitum*, in a temperature/humidity controlled-room maintained on 12 h light/dark cycles.

All surgical procedures were performed as previously described by our group (Teixeira et al., 2017; Mendes-Pinheiro et al., 2018). Succinctly, the PD model was induced by a unilateral stereotaxic injection of 20 mM 6-OHDA dissolved in saline containing 0.2 mg/mL ascorbic acid. The 6-OHDA group was injected with 2 μL of 6-OHDA in the right medial forebrain bundle (MFB; *n* = 15), and the sham group (*n* = 9) received an equal volume of vehicle solution in the same brain region. Five weeks later, some animals received hBMSCs transplants and others hBMSCs secretome in the SNpc and striatum (four different sites), and the groups were divided as follows: 6-OHDA control group (Neurobasal-A medium; *n* = 5), Sham group (sterile saline; *n* = 9), hBMSCs transplants (*n* = 4), and hBMSCs secretome (*n* = 6). The 6-OHDA-control group received 4 μL of 100 × concentrated Neurobasal-A medium in the SNpc and 2 μL in each coordinate of striatum. The same volumes and concentrations were injected in the animals that received hBMSCs secretome. Cell transplanted groups received 200,000 cells (suspended in serum-free medium) in SNpc and 200,000 cells in the striatum divided equally for each coordinate (Mendes-Pinheiro, 2016). The coordinates used in this work were done according to the rat brain atlas Paxinos and Watson (2007) as already reported (Teixeira et al., 2017; Mendes-Pinheiro et al., 2018).

### Behavioral Testing

**Figure 1:**
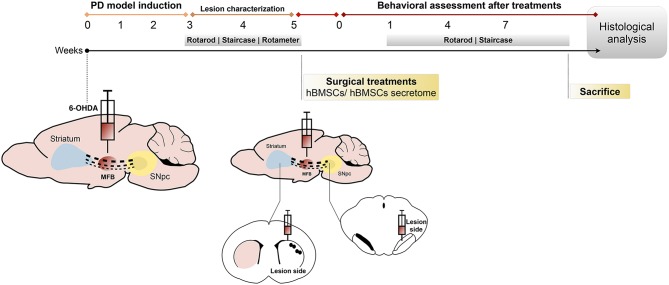
Design of the *in vivo* experiments. The animals were unilaterally injected with 6-OHDA directly into the MFB. After 3 weeks, animals were behaviorally characterized (through rotarod, staircase and rotameter tests) to validate the model. At week 5, 6-OHDA-lesioned animals received hBMSCs transplants or hBMSCs secretome in the SNpc and striatum. Behavior analysis was performed 1, 4, and 7 weeks after treatments, using the rotarod and staircase tests, and posteriorly sacrificed to proceed with the histological analysis.

Three weeks after 6-OHDA injections, animals were submitted to a first behavioral analysis for PD model characterization. In order to address the impact of hBMSCs transplants or hBMSCs secretome injections on motor performance of the animals, behavioral assessment was performed at 1, 4, and 7 weeks after treatments (Figure 1). Firstly, rats were tested in the rotarod apparatus (3376-4R, TSE systems, USA) to evaluate their motor coordination and balance as previously described (Monville et al., 2006). Then, to assess the fine motor control, the staircase test was performed using the protocol developed by Montoya et al. (1991).

Last, in order to estimate the dopaminergic denervation and to select the animals that were truly lesioned upon 6-OHDA injections, the rotameter test using apomorphine was performed as previously described (Carvalho et al., 2013).

### TH Immunohistochemistry and Quantification in SNpc and Striatum

To further evaluate the degree of dopaminergic preservation, immunohistochemical staining for TH was performed. For that, animals were sacrificed after 13 weeks (including lesion characterization and behavioral assessment after treatments) with sodium pentobarbital (Eutasil, 60 mg/kg, i.p.; Ceva Saúde Animal, Portugal) and perfused through the ascending aorta with 4% PFA in PBS. Brains were post-fixed in 4% PFA during 24 h, followed by 30% sucrose in PBS for 1 week. Coronal striatal and mesencephalon sections were obtained using a vibratome (VT1000S, Leica, Germany) with 50 μm of thickness. For TH staining, the endogenous peroxidases activity was stopped in the free-floating sections using 3% hydrogen peroxidase for 20 min at RT, followed by permeabilization in 0.1% PBS-T for 10 min (three times) and blockage in PBS 10% NBCS. After this, the sections were incubated with anti-TH (rabbit polyclonal IgG, 1:2,000; Merck Millipore) overnight at 4°C. In the next day, sections were incubated with a biotinylated secondary antibody (goat anti-polyvalent, TP-125-BN, ThermoFisher Scientific), followed by strepptavidine-peroxidase solution (TP-125-HR, ThermoFisher Scientific) for 30 min at RT. Antigen revelation was done using of 3,3′-diaminobenzidine tetrahydrochloride (DAB; D5905, Sigma) (25 mg DAB in 50 ml Tris–HCl 0.05 M with 12.5 μl H_2_O_2_, pH 7.6). Sections were mounted on slides, and after 24 h drying in the dark, they were counter-colored with thionin (Mendes-Pinheiro, 2016).

The estimation of the TH-positive cells' preservation was done by counting the total number of DA neurons in the SNpc and by densitometry analysis of the fibers in the striatum, in both hemispheres, as already described in detail (Teixeira et al., 2017). Four slices per animal were randomly chosen for evaluation and all the analysis was performed under blind conditions. Data is presented as the percentage (%) of remaining TH-positive cells in the lesioned side compared to the control side (intact side) for both regions.

### Untargeted Mass Spectrometry Proteomic Analysis

In order to characterize the hBMSCs secretome we performed a non-targeted proteomic analysis based on a combined mass spectrometry (MS) approach as previously described (Mendes-Pinheiro et al., 2018). Only proteins with at least two confidently identified peptides were considered as positive identifications. Peptide's confidence was assessed by a False Discovery Analysis (FDR) and a minimum of 99% confidence (<1% FDR) was used to select the peptides. Three biological replicates of secretome were processed, and proteins that were just identified in a single biological replicate were not considered for analysis. The MS proteomics data have been deposited to the ProteomeXchange Consortium via the PRIDE (Perez-Riverol et al., 2019) partner repository with the dataset identifier PXD014887. The final list of proteins (Supplementary Material) was used for gene ontology characterization (levels 2 and 3) using PANTHER (Mi et al., 2013), regarding molecular function, protein class and signaling pathways. A levels 4 and 5 gene ontology analysis was performed through an over-representation analysis using the ConsensusPathDB (Kamburov et al., 2013), for molecular function. Protein complex-based gene sets were determined using the same software, and assuming a minimum complex size of 2, minimum overlap with input list of 2, and *p*-value cutoff of 1%. The two most represented protein complexes are shown, based on the *p*-value for association.

### Statistics

A confidence interval of 95% was assumed for all statistical tests. The assumption of normality was tested for all continuous variables through evaluation of the frequency distribution histogram, the values of skewness and kurtosis and through the Shapiro-Wilk test. The assumption of homoscedasticity was tested through Levene's test. Both assumptions were met by all tested continuous variables. All continuous data is shown as the mean as mean ± SEM. For the comparison of proportions between different groups, a Chi-square test was performed, followed by a z-test for the comparison of independent proportions with the Bonferroni correction. For the comparison of means between two groups, a Student's *t*-test for independent samples was used, when data was continuous; for discrete data, a Mann-Whitney U test was carried out. For the evaluation of mean differences in samples with one independent and one repeated measures variable, a mixed design ANOVA was carried out, with Tukey's *post-hoc* test for pairwise comparison of the independent variable. Statistical analysis was performed using IBM SPSS Statistics ver.24 (IBM Co., USA) and graphic representation using GraphPad Prism ver.7.0c (GraphPad Software; La Jolla, USA).

## Results

### Effects of hBMSCs Secretome on Neuronal Differentiation *in vitro*

**Figure 2:**
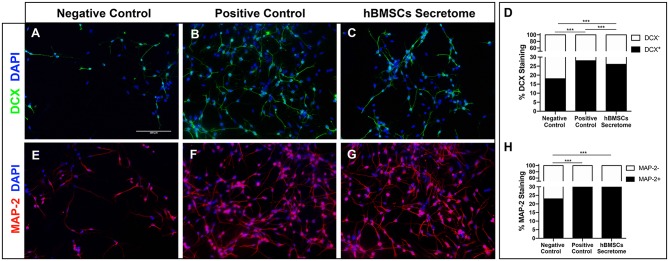
Effect of hBMSCs secretome in the differentiation of hNPCs *in vitro*. **(A–C)** Representative microphotographs of DCX staining in hNPCs and **(D)** respective quantification of the proportion of stained cells, as marker of immature neurons [Chi-squared, $\chi_{\left(2\right)}^{2}$ = 79.862, *p* < 0.0001]. A total number of 1,906 cells were scored for the negative control and a minimum of 13,000 for the other conditions, across two independent experiments. **(E–G)** Representative microphotographs of MAP-2 staining in hNPCs and **(H)** respective quantification of the proportion of stained cells, as a marker of mature neurons. A total number of 1,620 cells were scored for the negative control, and a minimum of 23,000 for the other conditions, across two independent experiments [Chi-squared, $\chi_{\left(2\right)}^{2}$ = 113.367, *p* < 0.0001]. Nuclei were stained with DAPI. ***p < 0.0001 (Scale bar: 100 μm).

hNPCs grow as neurospheres in the presence of PPRF-h2 serum-free medium as previously described (Baghbaderani et al., 2010; Teixeira et al., 2015). Typically, upon removal of their growth medium hNPCs lose their neurosphere-like conformation, adhere and spontaneously start to differentiate. The differentiation of hNPCs was further confirmed by immunocytochemistry analysis for DCX and MAP-2, after 5 days in culture, staining for immature and early stage mature neurons, respectively. As expected, hNPCs with basal media (negative control) were unable to differentiate, while cells with differentiation medium (positive control) showed positive differentiation for both MAP-2 and DCX neurons (Figures 2A–D). Interestingly, the hBMSCs secretome was also able to increase the proportion of differentiated neurons, as shown by an increase in MAP-2 staining, similar to differentiation rates of standard neuronal differentiation media-treated cells (Figures 2E–H).

### Injection of hBMSCs Secretome Attenuates the Fine Motor Deficits of 6-OHDA-Lesioned Animals

**Figure 3:**
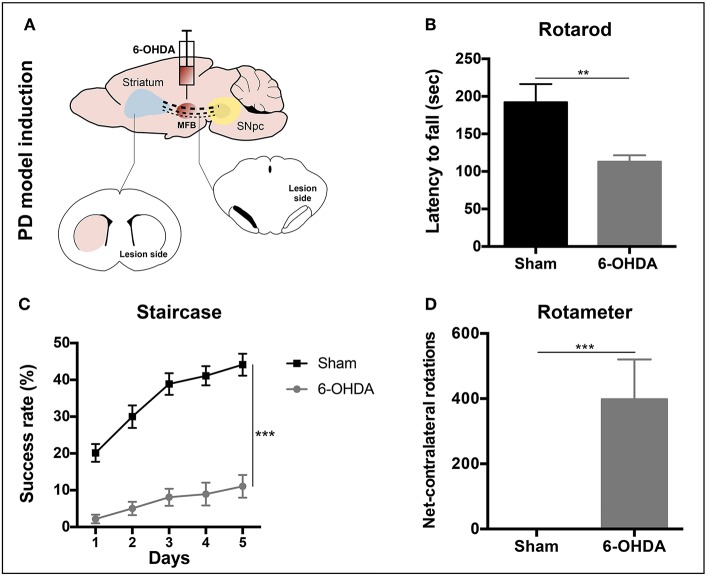
PD model validation. **(A)** Animals received a unilateral injection of 6-OHDA into the MFB in order to induce PD-like symptoms. **(B)** Latency to fall was measured in the accelerating rotarod test, demonstrating that 6-OHDA-injected animals presented significant motor impairment in motor coordination and balance [Student's *t*-test, *t*_(19)_ = 3.849, *p* < 0.01, Sham: *n* = 7, 6-OHDA: *n* = 14]. **(C)** Deficits in forelimb use and skilled motor function were also observed after the staircase test evaluation [Mixed design ANOVA, *F*_group_(1,22) = 76.290, *p* < 0.0001, Sham: *n* = 9, 6-OHDA: *n* = 15]. Data is presented as mean ± SEM. **(D)** Rotameter test revealed that 6-OHDA-injected animals exhibited an intense turning behavior, showing a clear decline of the dopaminergic system (Mann-Whitney, *U* = 0, *p* < 0.0001, Sham: *n* = 9, 6-OHDA: *n* = 15). Data is presented as median ± IQR. **p < 0.01, ***p < 0.001.

**Figure 4:**
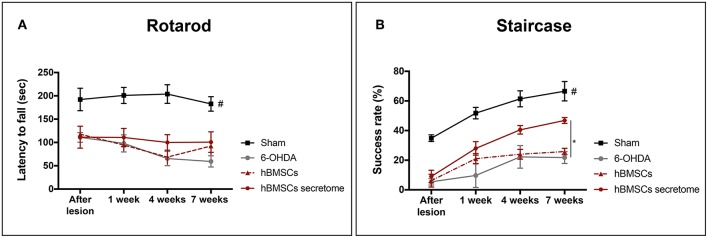
Effects of hBMSCs transplants and hBMSCs secretome injections on the motor performance of 6-OHDA-lesioned animals at 1, 4, and 7 weeks after treatments. **(A)** No differences were found in the 6-OHDA-lesioned animals' motor coordination and balance after hBMSCs secretome administration [Mixed design ANOVA, *F*_Group_(3,17) = 28.875, *p* < 0.0001, Sham: *n* = 7, 6-OHDA: *n* = 5, hBMSCs: *n* = 4, hBMSCs secretome: *n* = 5]. **(B)** In the staircase test, used to evaluate the fine motor movements, animals treated with hBMSCs secretome showed a significant increase in the success rate of eaten pellets when compared to the untreated group 6-OHDA [Mixed design ANOVA, *F*_Group_(3,20) = 22.804, *p* < 0.0001, Sham: *n* = 9, 6-OHDA: *n* = 5, hBMSCs: *n* = 4, hBMSCs secretome: *n* = 6]. Data is presented as mean ± SEM. *p < 0.05; Sham animals statistically different from all the other groups: ^#^*p* < 0.0001.

Three weeks after 6-OHDA injections into the MFB (Figure 3A), rats were behaviorally characterized in order to evaluate PD-like symptoms. To access the motor function of the animals, rotarod and the staircase tests were performed. Motor coordination and balance, assessed by the rotarod test, was found to be impaired in animals injected with 6-OHDA (Figure 3B). In the staircase test, performed to assess the forelimb use and skilled motor function, it was also observed that the 6-OHDA-injected animals were significantly affected when compared to sham animals (Figure 3C). To evaluate the level of DA depletion the rotameter test was performed at the end of rotarod and staircase tests, and an intense turning behavior after apomorphine administration was observed in 6-OHDA-injected animals when compared with sham group (Figure 3D) (Mendes-Pinheiro, 2016). Then, to address the effects of either hBMSCs transplants or hBMSCs secretome injections, as well as comparing both treatments, the motor performance of the animals was evaluated in three different time-points after treatments (1, 4, and 7 weeks) following the same timeline as previsouly reported elsewhere (Teixeira et al., 2017; Mendes-Pinheiro et al., 2018). Regarding the rotarod test, motor coordination and balance of the 6-OHDA-lesioned animals was unchanged upon hBMSCs cell transplantation or secretome administration (Figure 4A). On the other hand, in the staircase test, used to assess the fine motor movements, animals treated with hBMSCs secretome showed a significant increase in the success rate of eaten pellets when compared to the untreated group (Figure 4B). Moreover, the treatment with hBMSCs transplants was not able to improve the motor function of the animals in the staircase test when compared to the untreated group (Figure 4B).

### Injection of hBMSCs Secretome Protects Against TH Damage in SNpc and Striatum

**Figure 5:**
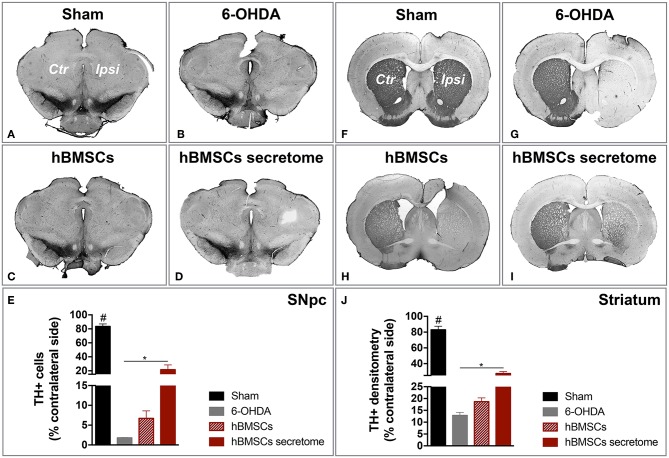
Injection of hBMSCs secretome protects against DA neurons damage. **(A–E)** The TH-positive cells were counted in the entire SNpc, and we observed that hBMSCs secretome injections was able to minimize the TH loss when compared to the untreated group 6-OHDA [One-way ANOVA, *F*_(3, 23)_ = 84.541, *p* < 0.0001, Sham: *n* = 9, 6-OHDA: *n* = 5, hBMSCs: *n* = 4, hBMSCs secretome: *n* = 6]. **(F–J)** The same effect was also detected in the striatum, by measuring TH-positive fibers through densitometry analysis [One-way ANOVA, *F*_(3, 20)_ = 110.564, *p* < 0.0001, Sham: *n* = 6, 6-OHDA: *n* = 5, hBMSCs: *n* = 4, hBMSCs secretome: *n* = 6]. Data is presented as mean ± SEM. *p < 0.05; Sham animals statistically different from all the other groups: ^#^*p* < 0.0001.

After histological analysis in the SNpc and striatum, we observed that the treatment with hBMSCs secretome was able to signifcantly minimize the dopaminergic loss upon 6-OHDA administration, which was not verified with hBMSCs transplanted group. In fact, TH staining revealed a significantly higher number of TH-positive cells in the SNpc when compared to the untreated group 6-OHDA (Figures 5A–E). Likewise, the same difference in the striatum was observed, by measuaring TH-positive fibers through densytometry analysis (Figures 5F–J). These finding are particularly important since they were obtained with a one-time administration of hBMSCs secretome, which produced effects over the course of the following 7 weeks. Moreover, it also important to highlight the differences obtained when compared to the hBMSCs cell transplanted group, which has been considered for many years one of the go-to strategies in stem cell base approaches for PD regenerative medicine.

### Characterization of Protein Sets That Constitute the Secretome of hBMSCs Secretome

**Figure 6:**
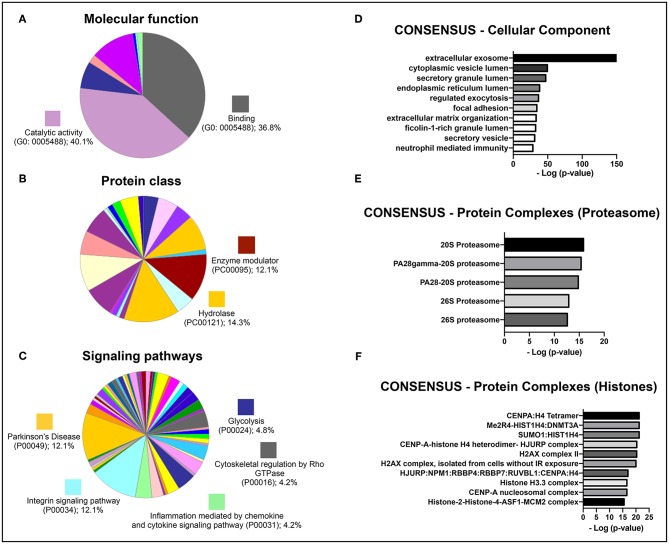
Gene ontology analyses of the identified proteins in a proteomic analysis of the hBMSCs secretome from three independent donors. Categorization of the most represented **(A)** molecular functions, **(B)** protein classes, and **(C)** signaling pathways, using the PANTHER software (gene ontology levels 2 and 3). Results are shown as the percentage of proteins that are part of each individual GO category. Categorization of the enrichment of **(D)** cellular components, **(E)** elements of the proteasome and **(F)** elements of histones, using the ConsensusPathDB software (gene ontology levels 4 and 5). Results are shown as the –log of the *p*-value for the enrichment of each individual category.

To identify potential therapeutic molecules in the hBMSCs secretome that could be involved in the strong response of the secretome-injected animals, the latter was characterized through a non-biased proteomic analysis based on a combined mass spectrometry (MS) approach (Mendes-Pinheiro, 2016). We were able to identify 279 proteins (according to the UniProtKB/Swiss-Prot classification) in the hBMSCs secretome, common to all replicates, from which 426 were detected based on 2 or more peptides, and therefore used in further analyses. An initial gene ontology analyses (levels 2 and 3) was carried out using PANTHER, in order to broadly characterize the protein types presented in the hBMSCs secretome. Regarding molecular function, more than 80% of identified proteins were either allocated in the binding or catalytic activity categories (Figure 6A), which is in accordance with the fact that this is a secreted media (Teixeira et al., 2016; Mendes-Pinheiro et al., 2018). In agreement, classification by protein class revealed that hydrolases and enzyme modulators were in the top three categories (Figure 6B). Regarding the signaling pathways that are mostly represented in the secretome, Parkinson's disease-related proteins was one of the top categories (Figure 6C), which suggests that the secretome contains protein elements that might be beneficial for PD context. A more detailed gene ontology analysis (levels 4 and 5) was then performed using the ConsensusPathDB. Interestingly, the most represented category of cellular components observed was extracellular exosomes (Figure 6D). When it comes to the mostly represented protein complexes, we identified elements from the proteasome (Figure 6E) and from histones (Figure 6F) as enriched in the secretome. This analysis provides interesting clues on the relevance of the composition of hBMSCs secretome in the context of PD.

## Discussion

The main goal of this study was to determine the impact of the injection of the secretome-derived from hBMSCs as a potential therapy for PD, when compared to what is considered the current gold standard in the field of cell-based therapies for regenerative medicine, that is the stem cell-based transplantation approaches.

PD is characterized by an extensive loss of DA neurons in the SNpc and their terminals in the striatum, resulting in debilitating motor problems (Przedborski, 2017). Therefore, in the *in vivo* experiments, in order to cause dopaminergic degeneration, was used a rat PD model induced by 6-OHDA injection into the MFB (Carvalho et al., 2013; Mendes-Pinheiro, 2016). As in line with previous reports (Teixeira et al., 2017; Mendes-Pinheiro et al., 2018), here we were able to successfully establish the model. As shown in the rotameter test (Figure 3D), 6-OHDA-lesioned animals displayed a strong turning behavior when compared to the sham group (injected with saline), indicating a clear magnitude of the lesion. We also verified that the motor function of these animals was affected, since they presented deficits in motor coordination and balance, as well as in the skilled motor function, addressed by the rotarod and staircase tests, respectively (Figures 3B,C). Regarding the effects of the treatments, we observed that neither the hBMSCs transplants nor secretome injections were able to improve the animals' motor coordination and balance in the rotarod test (Figure 4A). It is important to state that the rotarod test is commonly used in this context, however, it has also been described that is not the most sensitive test to use in models that present basal ganglia dysfunctions (Magen and Chesselet, 2010; Mann and Chesselet, 2014). Moreover, since we used the accelerating protocol during 4 consecutive days (4 trials per day), the fatigue of the animals could explain in part the results obtained. On the other hand, the fine motor skill task has been used in different models of PD, and it was shown to be very suitable to detect striatum/nigrostriatal bundle unilateral lesions (Baird et al., 2001). Indeed, when the forelimb reaching and grasping abilities were assessed using the staircase test, we observed that the injection of hBMSCs secretome improved the success rate of eaten pellets of the treated animals when compared to the untreated group 6-OHDA (Figure 4B). Additionally, rats injected with hBMSCs secretome showed better preservation in the number of TH-positive neurons in the SNpc, as well as an increase in the density of TH-positive fibers in the striatum (Figure 5). These histological outcomes nicely correlate with positive functional improvements that we observed in the fine motor movements for the animals treated with secretome. On the other hand, such evidences were not observed in hBMSCs-transplanted animals, probably due to the low survival rate of cells upon transplantation. We performed human nuclear antigen staining for hBMSCs detection (*data not shown*), and we were not able to observe the presence of the grafts on both SNpc and striatum. In fact, it has been shown that the MSCs survival is minimal and the implantation time of these cells is usually too short to have an effective impact (Vizoso et al., 2017).

In the past few years MSCs has been widely studied as therapeutical agents in different pathological conditions of the central nervous system (CNS), including PD (Teixeira et al., 2013; Salgado et al., 2015; Konala et al., 2016). Primary studies showed that transplanted MSCs were able to repair injured adult mesenchymal tissues; thereafter, some authors also reported the capacity of MSCs to transdifferentiate into ectodermal-derived cells (Donega et al., 2014; Takeda and Xu, 2015; Bagher et al., 2016). While these studies were accompanied with some controversy throughout the years, robust data have been demonstrating that the paracrine activity of MSCs could have a critical role in its beneficial effects. In fact, MSCs are able to secrete a wide spectrum of elements with strong immunomodulatory properties, which are also able to inhibit apoptosis, enhance angiogenesis and promote neuronal survival and differentiation (Marote et al., 2016). This is the first study comparing directly hBMSCs secretome injections with hBMSCs transplantation. Nonetheless, other authors correlated motor improvements and DA neurons' protection with the secretion and local increase of different growth factors after MSCs transplantation (Sadan et al., 2009; Cova et al., 2010; Wang et al., 2010; Cerri et al., 2015), supporting the secretome theory.

To explore the possible underlying mechanisms or key molecules behind the secretome effects, we characterized the latter using a MS proteomic approach. Interestingly, when we evaluated cellular components at a more detailed level, we observed a very large enrichment of proteins that are part of exosomes. Moreover, this category of proteins was clearly overrepresented when compared with the other top hits. In fact, some studies demonstrate that the administration of MSCs-derived exosomes was able to rescue tissue function in different disease/injury contexts and to induce beneficial *in vitro* effects, mainly mediated by exosomal-enclosed microRNAs (miRNAs) (Marote et al., 2016; Vilaça-Faria et al., 2019). For instance, Jarmalavičiute et al. (2015), using a 6-OHDA 3D culture model, showed that dental pulp MSCs-derived exosomes rescue DA neurons from cell death. Hence, the beneficial effects of the hBMSCs secretome that observed in this work could be mediated by a similar exosome-dependent mechanism. Additionally, other studies demonstrated the potential of exosomes produced by MSCs on neuronal differentiation. For instance, Lee et al. (2014) showed that BMSCs had the capacity to the deliver miRNAS, namely miR-124 and miR-145, to NPCs and astrocytes, impacting cell differentiation and increasing the expression of glutamate transporters. Another study, exposing cortical neurons to MSCs-derived exosomes, showed an improvement in neurite outgrowth, by increasing both neurite branch and total length, and attributing the effects to the transference of miR-133b to neural cells (Xin et al., 2012). Similarly, Lopez-Verrilli et al. (2016), showed that menstrual MSCs-derived and BMSCs exosomes promote neurite growth in cortical neurons and dorsal root ganglia neurons, respectively. This is also in line with our results, since secretome-treated hNPCs showed and increase proportion of MAP-2 staining. Moreover, another interesting study using a 6-OHDA mice PD model, reported that mimic-miR-124 increased neurogenesis in the subventricular zone (SVZ) which was correlated with significant behavioral improvements (Saraiva et al., 2016). Altogether, this evidence indicates that besides dopaminergic survival, the modulation of neurogenesis may also have influence in the recovery of PD, and could be one of the reasons behind the observed secretome-effects.

The precise cause of PD remains elusive, but compelling evidence spotlight the ubiquitin proteasome system (UPS) as a key feature in PD pathogenesis (Bentea et al., 2017). This connection was supported due to genetic mutations in the *PRKN* and UCHL1 genes, with critical roles in the UPS system, with familial parkinsonism (Kitada et al., 1998; Summers et al., 1998). In this follow up, McNaught et al. (2001) observed a significant decrease of proteasomal core subunits in the SN of sporadic PD brains. Furthermore, some studies showed that proteasomal inhibitors like lactacystin and proteasome inhibitor 1 (PSI) leads to dopaminergic cell death *in vitro* and after brain injections (McNaught et al., 2002a,b; Matsui et al., 2010; Xie et al., 2010). Although it is not part of the UPS, overexpression of wild-type or mutant α-synuclein both *in vitro* and *in vivo* was also shown to impede proteasome function (Stefanis et al., 2001; Tanaka et al., 2001; Chen et al., 2006). With UPS dysfunction being one of the pathophysiological hallmarks of PD, and since one of more enriched protein networks in the hBMSCs secretome was the proteasome, we can speculate that the restoration of UPS-dependent proteostasis could be one of the main mechanism responsible for the phenotypical improvement we observed.

Finally, we also observed that histones are one of the mostly represented protein complexes in hBMSCs secretome. Indeed, histone modifications have been linked with the development, differentiation and maintenance of DA neurons (van Heesbeen et al., 2013). Park and co-workers (Park et al., 2016) demonstrated an increase of histone acetylation in DA neurons of PD patients when compared to healthy individuals. Besides that, using the MPTP model both *in vitro* and *in vivo*, the same authors showed a decrease of multiple histone deacetylases (HDACs), as well as in the midbrain tissues of human PD patients (Park et al., 2016). In cellular models, either dieldrin or paraquat were shown to induce histone acetylation (Song et al., 2010, 2011). In addition, Goers et al. (2003) showed in culture an interaction between histones and α-synuclein via complex formation, as well as, that α-synuclein fibrillation was intensely enhanced in the presence of histones. Another study, using two different PD models, demonstrated that DA reduction and following levodopa treatment were related with intense alterations in post-translational modifications histones in the striatum (Nicholas et al., 2008). Altogether, these findings suggest that chromatin remodeling may have an important role in PD pathogenesis.

Additionally, previous work from our already showed that hBMSCs is a source of numerous neuroregulatory molecules with neuroprotection character (Pires et al., 2016). For instance, similarly to the above referred work, we found in our proteomic analysis, important proteins known as anti-oxidant agents, such as DJ-1, TRXR1, and PRDX1 that have been shown to counteract dopaminergic cell death in different PD models as reviewed by Pires et al. (2016). Also PEDF, it is known for its neurotrophic properties but also for neuroprotective roles in different PD toxin models (Falk et al., 2009). This neuroprotective effects have been linked to the capacity for induce the activation of nuclear factor NF-κB signaling cascade, which in turn induces the expression of genes that are essential to neuronal protection and survival, such as BDNF and GDNF (Falk et al., 2010). Moreover, other proteins like Galactin-1 and Cystatin C have also been associated with anti-apoptotic effects in *in vitro* models of PD (Pires et al., 2016).

Although candidate molecules and possible mechanisms are under investigation, further detailed studies are needed to carefully define which players may be responsible for the hBMSCs secretome-mediated effects. In the future, we intend to conduct studies, both *in vitro* and *in vivo*, regarding the identification of some of these molecules and their roles in the context of PD. In conclusion, the present findings support hBMSCs secretome treatment as a novel therapeutic strategy for PD. Moreover, we have also shown that its application in a well-described rat model of PD is more effective than the traditional cell transplantation approaches, which have been considered one of the most preferred strategies in the field of stem cell-based PD regenerative medicine for several years. However, additional work is still necessary in order to improve the applicability of this approach.

## Data Availability Statement

The dataset PXD014887 for this study can be found in the ProteomeXchange Consortium via the PRIDE partner repository (http://www.ebi.ac.uk/pride).

## Ethics Statement

The animal study was reviewed and approved by Direção Geral de Alimentação e Veterinária (ID: DGAV28421) and Ethical Subcommittee in Life and Health Sciences (SECVS; ID: SECVS-008/2013, University of Minho).

## Author Contributions

BM-P designed and performed most of the experiments, collected and analyzed the data, and drafted the manuscript. SA and BM performed and collected the data regarding the proteomic analysis. JD contributed to the proteomic and statistical analysis, and helped with the manuscript writing. AM helped with the animal experiments and with the manuscript writing. LB provided the study material and helped with the interpretation of the results. FT performed the stereotaxic surgeries, helped with the data interpretation, and with the manuscript writing. AS conceived and financially support the study, participated in its design and coordination, and critically read the manuscript. All authors read and approved the final manuscript.

### Conflict of Interest

The authors declare that the research was conducted in the absence of any commercial or financial relationships that could be construed as a potential conflict of interest.
